# Relined fiberglass posts: influence of resin opacity on the bond strength to intraradicular dentin

**DOI:** 10.1590/0103-6440202405746

**Published:** 2024-07-22

**Authors:** Ana Teresa Maluly-Proni, Mariana Elias Queiroz, Matheus Henrique Gomes, Thaís Yumi Umeda Suzuki, Bruna de Oliveira Reis, Paulo Henrique dos Santos

**Affiliations:** 1 Department of Preventive and Restorative Dentistry, Araçatuba School of Dentistry, São Paulo State University, UNESP, Araçatuba, São Paulo, Brazil.; 2 Department of Dental Materials and Prosthodontics, Araçatuba School of Dentistry, São Paulo State University, UNESP, Araçatuba, São Paulo, Brazil.; 3 Department of Restorative Dentistry, Federal University of Minas Gerais, UFMG, Belo Horizonte, MG, Brazil.; 4 Dental Research Institute, University of Toronto, Faculty of Dentistry - UofT, Toronto, ON, Canada.

**Keywords:** Resin cements, dentin, post and core technique

## Abstract

This study aimed to evaluate the bond strength between relined fiberglass posts with different composite resin opacities in different thirds of the intraradicular dentin. Thirty single-rooted premolars were endodontically treated and divided into three groups (n=10): fiberglass posts relined with translucent composite resin, fiberglass posts relined with opaque composite resin, and fiberglass posts without relining. After cementation of the posts, the specimens were cut perpendicular to the long axis of the tooth in slices of approximately 1.3 mm of each third to be analyzed (cervical, middle, and apical) and then subjected to the push-out test. The bond strength (MPa) data were subjected to Shapiro-Wilk normality tests and two-way repeated measures analysis of variance, considering the experimental groups and different regions as study factors. Tukey’s post-hoc test (p<.05) was applied for comparisons between the groups. In the cervical third, higher values of bond strength were found for the group relined with translucent resin, with a statistically significant difference for the other groups. In the other regions, both opaque and translucent resins showed similar results, but both showed higher values of bond strength compared to fiberglass posts without relining. The bond strength of fiberglass posts to different thirds of intraradicular dentin is influenced by composite resin relining as well as the opacity of the resin. The use of translucent composite resin is a more effective alternative for fiberglass posts relining.

## Resumo

Este estudo teve como objetivo avaliar a resistência de união entre pinos de fibra de vidro anatomizados com resinas compostas de diferentes opacidades em diferentes terços da dentina intrarradicular. Trinta pré-molares uniradiculares foram tratados endodonticamente e divididos em três grupos (n=10): pinos de fibra de vidro anatomizados com resina composta translúcida, pinos de fibra de vidro anatomizados com resina composta opaca e pinos de fibra de vidro sem anatomização. Após a cimentação dos pinos, os corpos de prova foram cortados perpendicularmente ao longo eixo do dente em fatias de aproximadamente 1,3 mm de cada terço a ser analisado (cervical, médio e apical) e posteriormente submetidos ao teste de push-out. Os dados de resistência de união (MPa) foram submetidos a testes de normalidade de Shapiro-Wilk e ANOVA dois fatores para análises repetidas, considerando os grupos experimentais e diferentes regiões como fatores de estudo. O teste post-hoc de Tukey (p=0,05) foi aplicado para comparações entre os grupos. No terço cervical foram encontrados maiores valores de resistência de união no grupo com anatomização com resina translúcida, com diferença estatisticamente significativa para os demais grupos. Nas demais regiões, tanto as resinas opacas quanto as translúcidas apresentaram resultados semelhantes, mas ambas apresentaram valores de resistência de união superiores aos pinos de fibra de vidro sem anatomização. A resistência de união dos pinos de fibra de vidro aos diferentes terços da dentina intrarradicular é influenciada pela anatomização da resina composta, bem como pela opacidade da resina. A utilização de resina composta translúcida é uma alternativa mais eficaz para a anatomização de pinos de fibra de vidro.

## Introduction

Fiberglass posts are increasingly gaining acceptance among dental clinicians for the restoration of teeth with significant crown structure loss caused by caries, trauma, or early endodontic procedures. In these clinical situations, fiberglass posts have emerged as the preferred intracanal retainer option when well indicated. ^1^. The material has become popular due to its distinctive characteristics, including its esthetic capacity and mechanical properties similar to dentin, which favors a more uniform dissipation of tension on the remaining tooth structure ^1^ ^,^ ^2^ ^,^ ^3^. Structurally, fiberglass posts allow bonding to root dentin through adhesive bonding techniques, and owing to its physical and mechanical properties, reduce stress concentration ^4^ and incidence of root fractures ^1^ ^,^ ^4^.

Unlike metallic posts, where the most common failure is associated with residual root fracture, fiberglass post restorations are mostly linked to debonding failures ^3^ ^,^ ^5^ ^,^ ^6^. Some factors can contribute to this adhesive failure, including difficulties in controlling moisture and residues in intraradicular dentin, a high endodontic C-factor, the anatomy of the root canal, histology of the intraradicular dentin, and difficulty in accessing the middle and apical thirds of the canal, which impairs the correct execution of the cementation steps ^6^ ^,^ ^7^. Furthermore, as fiberglass posts are prefabricated material, they lack proper adaptation to the root canal walls. In clinical situations where the root canal is large or exhibits irregular shapes, achieving adequate adaptation of the post to canal walls becomes challenging, leading to a thick cementation line ^2^. The increased thickness of this cement layer raises the likelihood of bubble formation, consequently resulting in adhesive failure ^5^.

A suggested technique for utilizing fiberglass posts in wider canals involves employing relined posts with composite resin. This method enhances the adaptation of the post to the canal walls, resulting in improved retention and stability of the entire structure. Resin cements have a low inorganic filler content and exhibit suboptimal physical and mechanical properties when used in greater thickness ^5^. Furthermore, a primary factor contributing to the failure of resin-based composite materials is the stress generated at the adhesive interface due to polymerization shrinkage ^6^. This stress is influenced by factors such as resin volume, cavity configuration, and material elasticity modulus ^8^. The post-relining technique facilitates the creation of a thinner cement layer, diminishing the polymerization contraction of this material and mitigating stress on the adhesive interface between the relined post and the root canal walls ^2^ ^,^ ^6^.

The polymerization of resin materials is directly influenced by factors such as light irradiance, exposure time, composition, color, and translucency ^9^. Resin materials with greater opacity require higher light intensity for photoactivation ^9^ ^,^ ^10^. Limited penetration of light inside the conduit may impact the effectiveness of cement retainer systems ^7^, especially concerning the length of the retainer system, potentially leading to adhesion impairment ^9^ ^,^ ^10^. Consequently, in cases of fiberglass post relining, the color of the composite resin used could also influence the quality of light transmission through the post.

Ensuring the proper polymerization of the resin cement or composite resin is crucial. Therefore, adequate light transmission to the most apical portion of the conduit is essential to provide the necessary intensity for material activation. This, in turn, promotes enhanced adhesion of the post to the conduit ^11^. The current challenge lies in developing systems that enable the union of the dentin/cement/post trinomial in a compatible manner, overcoming the challenges of adhesion to intraradicular dentin ^12^ ^,^ ^13^.

Therefore, this study aimed to evaluate the bond strength between relined fiberglass posts with various composite resins of different opacities to different thirds of intraradicular dentin (cervical, middle, and apical). The following null hypotheses were tested: 1) relining fiberglass posts with different composite resins would not result in a difference in bond strength to dentin when compared with non-relined posts, and 2) there would be no difference in bond strength between the different thirds of the root canal, regardless of the anatomy of the fiberglass posts.

## Materials and methods

After obtaining approval from the Research Ethics Committee (#67674617.0.0000.5420), thirty single-rooted premolars were utilized and divided into three groups based on the retention system employed: fiberglass posts without relining, posts relined with translucent composite resin and posts relined with opaque composite resin (n=10).

**Figure 1:**
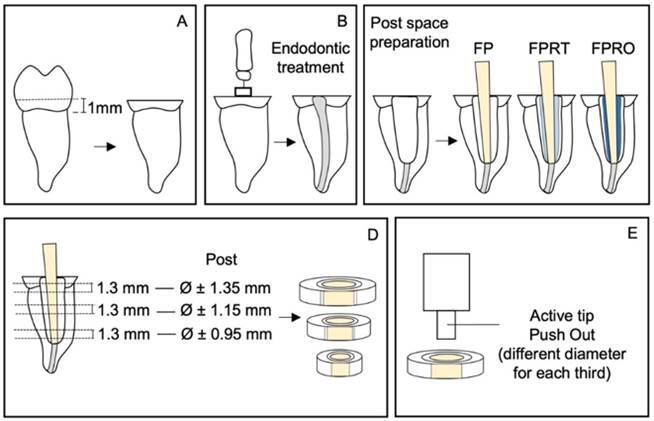
Specimen preparation scheme and push-out test. A- Removal of the anatomical crowns 1mm above the cementoenamel junction. B- Endodontic treatment and root canal filling. C- Preparation and cementation of the posts according to the division of the groups (FP, FPRO, and FPRT). D- Obtaining the slices corresponding to the three root thirds studied. E- Active tip used for the push-out test corresponding to the diameter of the post.

After selection, each tooth was bonded to an acrylic resin plate fixed to the support of a precision cutter (Isomet 1000, Buehler, Lake Bluff, Illinois, USA). The anatomical crowns of the teeth were removed through a 1-mm cross-section above the cementoenamel junction using a diamond cut-off wheel (Buehler, Illinois, USA) with dimensions 4 in X 0.012 in (102 mm diameter X 0.3 mm thickness) at low speed under refrigeration (Fig. 1A).

To simulate wide canals, the roots underwent endodontic treatment (Fig. 1B). Initial access to the canal was achieved using a No. 1 spherical bur (KG Sorensen - Medical Burs Industry, São Paulo, Brazil) at high speed under refrigeration. The working length was visually established by subtracting 1 mm from the total insertion length of a #10 K Flexofile file (Antaeos, Munich, BY, Germany) when it emerged at the apical foramen. Manual instrumentation was then performed along the entire working length up to a #45 K Flexofile file (Antaeos, Munich, BY, Germany). The canals were irrigated with 3 mL of 2.5% sodium hypochlorite using an irrigating syringe between each file change and during the instrumentation phase. After the final irrigation, the conduits were dried with absorbent paper cones and filled with gutta-percha cones (Dentsply-Maillefer, Oklahoma, USA) and Sealer 26 calcium hydroxide cement (Dentsply-Maillefer, Oklahoma, USA) using the lateral condensation technique. Following the removal of excess gutta-percha, the coronal access was sealed with Clip F temporary resin (Voco, Cuxhaven, Germany), and the specimens were stored in water at 37°C for 7 days ^14^.

The intraradicular retainer used was a White Post DC #1 fiberglass post (FGM, Joinville, SC, Brazil). Initially, a No. 1 Largo drill (Dentsply-Maillefer, Oklahoma, USA) was employed at low speed to remove the filling material at a depth of approximately 9 mm. The composition of the materials used in this study is described in Box 1. To standardize the wide canal, a drill corresponding to post number 3 was utilized, ensuring proper adaptation of the post to the canal (Fig. 1C). Following complete preparation, the posts were tested to verify the fit.

**Box 1:**
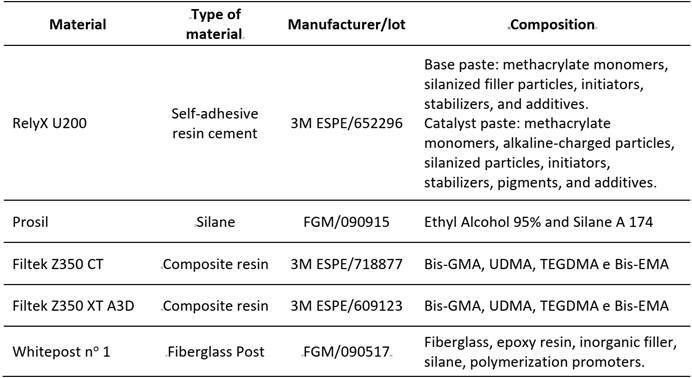
Materials used

### Specimen preparation

Group FP - Fiberglass posts without relining: The surface of the fiberglass post was cleaned with 70% alcohol, followed by the application of silane Prosil (FGM, Joinville, SC, Brazil) for 60 seconds and an air jet. Intraradicular dentin was irrigated with 2 mL of distilled water to remove gutta-percha remnants and maintain medium humidity. The canal was dried using an air jet and absorbent paper cones. The self-adhesive resin cement RelyX U200 (3M ESPE, Minnesota, USA) was manipulated and applied to the post and inside the canal with the aid of an endodontic file. Subsequently, the post was placed inside the canal, and excess cement was removed with a spatula. Finally, the resin cement was light-cured on the buccal, lingual, distal, and mesial faces of the coronal portion of the post for 20 seconds using a Valo Cordless LED light-curing device (Ultradent, Utah, USA) with a power of 1400 mW/cm^2^.

Group FPRT - Fiberglass posts relined with translucent composite resin: The surface of the fiberglass posts underwent the same treatment as described in Group FP. To reline the fiberglass posts with composite resin, the canal was lubricated with a water-soluble lubricating gel (KY; Johnson & Johnson). The posts were coated with translucent composite resin Filtek Z350 XT CT (3M ESPE, Minnesota, USA) and inserted into the canal. After removing excess material, the assembly was light-cured with a Valo Cordless LED (Ultradent Utah, USA) for 3 seconds. The assembly was then taken out of the root canal and light-cured for an additional 20 seconds on the buccal and palatal surfaces. Root canals were rinsed with a jet of distilled water for 30 seconds to remove the lubricant, followed by drying with air jets and absorbent paper cones. Relined posts were washed with distilled water and air-dried. Cementation of the relined fiberglass posts with translucent composite resin was carried out using RelyX U200 (3M ESPE, Minnesota, USA) self-adhesive resin cement, following the procedure described in Group FP.

Group FPRO - In Group FPRO, the same procedure for post-relining was performed, but this time using opaque resin Filtek Z350 XT A3D (3M ESPE, Minnesota, USA).

After the post-cementation process, all teeth were stored in distilled water at 37 °C in an incubator for 7 days.

### Bond strength evaluation by push-out test

To evaluate bond strength, slices of approximately 1.3 mm were initially obtained from the cervical, middle, and apical thirds of the samples using an Isomet 1000 precision cutter (Buehler, Illinois, USA) with a diamond blade at low speed, underwater cooling (Fig. 1D). The thickness of the specimens was measured with the aid of a digital caliper.

The specimens were positioned in an EMIC universal machine (Emic, PR, Brazil). In the upper portion, a metallic device was fixed with an active tip corresponding to the diameter of the post in different thirds of the root (cervical - 1.90 mm, middle - 1.5 mm, and apical - 1 mm). In the lower portion, the sample was placed on an acrylic resin support, with the most apical side facing upward. The metal rod in the upper portion of the machine was positioned close to the specimen, without touching it, ensuring that its active tip coincided with the center of the post to avoid tension in the surrounding root walls (Fig. 1E). A compressive load was applied in the vertical direction at a speed of 0.5 mm/min. The bond strength values obtained from the push-out test were calculated using the following formula ^15^:

Ru = F/a

where Ru (bond strength) is the division of the load required for failure (N) by the joint interface area (mm^2^). To determine the area of the joint interface, the following formula was used:

$$a\;=\;p(R1+R2)\surd {\left(R1-R2\right)}^{2}+h^{2}$$

where p = 3.14, R1= largest radius, R2= smallest radius, and h = cutting height. R1 and R2 were obtained by measuring the internal diameter of the base (major and smallest), which corresponded to the internal diameter between the walls of the root canal.

The bond strength (MPa) data were subjected to Shapiro-Wilk normality tests, two-way repeated measures analysis of variance (ANOVA, groups and root thirds), and Tukey post-test for comparison between groups (α=.05). All analyses were conducted using StatView Program, 5.0.1 version.

## Results

The two-way repeated measures ANOVA for the bond strength test showed statistically significant differences between the groups (p=.03), among the different regions studied (p<.0001), and in the interaction between the factors (p<.0001).

As shown in Table 1, in the cervical third, the highest bond strength values were observed in the group relined with translucent resin (10.00 ± 4.52 MPa), exhibiting a statistically significant difference compared to the groups relined with opaque resin (6.14 ± 2.04 MPa) and without relining (3.50 ± 2.43 MPa) (p < 0.05). Moving to the middle third, the highest bond strength values were found in the groups relined with opaque resin (6.99 ± 3.95 MPa) and translucent resin (5.13 ± 3.18 MPa), with no statistically significant difference between the groups (p > 0.05). Similarly, in the apical third, comparable bond strength values were observed for the groups relined with opaque resin (5.22 ± 3.20 MPa) and translucent resin (3.22 ± 2.23 MPa), with no statistically significant difference between them (p > 0.05).

**Table 1:** Push-out bond strength (MPa ± standard deviation) between fiberglass posts in the different thirds of intraradicular dentin

	FP	FPRT	FPRO
Cervical	3.50 ± 2.43 Ab	10.00 ± 4.52 Aa	6.14 ± 2.04 Ab
Middle	3.23 ± 2.29 Ab	5.13 ± 3.18 Bab	6.99 ± 3.95 Aa
Apical	1.65 ± 0.82 Ab	3.22 ± 2.63 Bab	5.22 ± 3.20 Aa

Means followed by distinct letters, capital letters in the column, and lowercase in the line, present a statistically significant difference (5%).

In the comparison between the studied thirds, the group relined with translucent resin exhibited a statistically significant difference, with the cervical third showing higher bond strength values (10.00 ± 4.52 MPa) compared to the middle and apical thirds. For the groups without relining and those relined with opaque resin, there was no statistically significant difference between the thirds (p > 0.05).

## Discussion

Due to their mechanical properties closely resembling those of dentin, fiberglass posts have an advantage over other types of intracanal retainers. This advantage lies in their ability to enable a more uniform dissipation of stress along the dental structure during the restoration of teeth with substantial loss of coronal structure ^2^. To attain clinical success in fiberglass post-cementation, it is crucial to achieve a thin and uniform thickness of cement in the canal. This ensures the most anatomically appropriate set possible. This step is regarded as exceptionally important and decisive, playing a crucial role in ensuring effective and lasting retention. Greater uniformity in the cementation line ensures better dissipation of forces to the intraradicular dentin ^3^. When the post's adaptation to the root canal walls is unsatisfactory, the cementation process results in a thick layer of resin cement. Since this material has a low elastic modulus, it leads to the creation of a zone with a concentrated load in this region ^16^. Furthermore, a greater volume of resin cement results in greater polymerization contraction of the material ^2^ ^,^ ^3^, which may contribute to failure in the adhesive process. Root canals do not have uniform anatomy; thus, relining of posts is a safe option for their adequacy, allowing a better adaptation of the post to the canal walls ^17^, reducing the resin cement layer, and providing less pressure at the dentin/cement and cement/post interface, thereby preventing the formation of bubbles and adhesion failures ^2^.

In this study, two variables were analyzed in the cementation of fiberglass posts: relining with translucent and opaque resins, and analysis of the different thirds of the root dentin (cervical, middle, and apical). Based on the results obtained, the use of composite resins with different opacities influenced the bond strength of the fiberglass post to intraradicular dentin (Table 1), thus rejecting the first null hypothesis of the study. Overall, the relining of fiberglass posts, regardless of the opacity of the material used, resulted in higher values of bond strength compared to those observed in the absence of relining (Table 1).

Some studies aimed to compare the cementation of relined posts with composite resin and fiberglass posts without relining ^2^ ^,^ ^5^ ^,^ ^18^ ^,^ ^19^. The findings of the current study are consistent with those of previous research, which noted higher bond strength values in the cementation of relined posts when contrasted with the bond strength associated with the conventional cementation of fiberglass posts ^5^ ^,^ ^18^ ^,^ ^19^. Prior studies ^2^ ^,^ ^18^ used human incisors and bovine teeth to simulate the cementation of conventional and relined fiberglass posts to compare the bond strength. The results revealed higher values in the relined groups. The authors attributed their results to the fact that the relining technique allowed for a reduction in the cement layer, as well as greater frictional retention.

The composite resins used for the relining of fiberglass posts depend directly on photoactivation, unlike the cementing agents used, which have dual activation (chemical and physical). The mechanical properties of resin materials are directly linked to their degree of conversion, and polymerization deficiency can affect the clinical performance of resin materials ^3^ ^,^ ^20^. The translucency of the composite resin used could have allowed greater activation of the resin cement, resulting in higher values of bond strength between the post and dentin, as observed in the cervical third of the FPRT group (Table 1).

As the depth increases, a greater amount of light is lost through the post/resin set, consequently reducing the activation of the light-cured portion of the resin cement due to reduced light transmission toward the root apex. The group that used opaque composite resins exhibited regularity in the values ​​of bond strength between the thirds, which could be explained by more homogeneous dissipation of light. This differs from the occurrence in the group that used translucent resin, where greater light transmission might have occurred in the cervical third, leading to a decrease in intensity in the most apical portion of the relined post (Table 1). Previous studies have shown that the color and opacity of the composite resin are factors that can interfere with the transmission of light through this material ^9^ ^,^ ^21^ ^,^ ^22^. A study ^21^ observed that the light irradiance at the bottom of the composite resin layer was greater for those with greater translucency. Another study ^22^ pointed out that resin polymerization efficiency could be influenced by the shade because lighter shade resin had higher hardness values on the lower surface. According to the authors, darker shade resins allow less light penetration because they are more opaque, which reduces the ability of light to penetrate. When determining the curing efficiency of different translucent and opaque shades of some commercial brands of composite resin ^23^, it was observed that for Filtek Z350 XT, the CT shade showed a higher degree of conversion on the upper and lower surfaces compared to those of A3E, A3B and A3D shades. This result was attributed to the low fraction of inorganic content in the CT resin or to the presence of alternative photoinitiators not described by the manufacturer.

However, to establish a better consensus on the application of this restorative technique, further studies are needed to evaluate variable lengths of relined fiberglass posts. Furthermore, to ensure better results in the rehabilitation process, future studies are needed to analyze the long-term effects of relining with different composite resins and the minimum energy density necessary to promote the adequate conversion of resin cement and enable stability of the bonding interface between fiberglass posts and dentin.

The limitations of this study include the non-reproducibility of all conditions found in the oral environment, such as the presence of saliva and stress caused by chewing efforts. Furthermore, the composite resins used to anatomize the post are from a single brand. More analyses using different brands and opacities should be carried out, in addition to clinical studies to explore the influence of oral cavity conditions.

Considering the results obtained, the bond strength of fiberglass posts is influenced by the anatomy of the composite resin as well as by its opacity. The use of translucent composite resin exhibited higher values of bond strength than opaque resin in the cervical third, proving to be an effective alternative for fiberglass posts relining.
